# High Prevalence of Klebsiella pneumoniae in European Food Products: a Multicentric Study Comparing Culture and Molecular Detection Methods

**DOI:** 10.1128/spectrum.02376-21

**Published:** 2022-02-23

**Authors:** Carla Rodrigues, Kathrin Hauser, Niamh Cahill, Małgorzata Ligowska-Marzęta, Gabriella Centorotola, Alessandra Cornacchia, Raquel Garcia Fierro, Marisa Haenni, Eva Møller Nielsen, Pascal Piveteau, Elodie Barbier, Dearbháile Morris, Francesco Pomilio, Sylvain Brisse

**Affiliations:** a Institut Pasteur, Université de Paris, Biodiversity and Epidemiology of Bacterial Pathogens, Paris, France; b Institute for Medical Microbiology and Hygiene, Austrian Agency for Health and Food Safety, Vienna/Graz, Austria; c Antimicrobial Resistance and Microbial Ecology Group, School of Medicine, National University of Ireland, Galway, Ireland; d Statens Serum Institut, Copenhagen, Denmark; e Istituto Zooprofilattico Sperimentale dell’Abruzzo e del Molise “G. Caporale”, Teramo, Italy; f Unité Antibiorésistance et Virulence Bactériennes, Université Claude Bernard Lyon 1 - ANSES, Lyon, France; g INRAE, UR OPPALE, Rennes, France; h Agroécologie, AgroSup Dijon, INRAE, Université Bourgogne Franche-Comté, Dijon, France; Institute of Biomedical Sciences, Universidade de São Paulo

**Keywords:** *Klebsiella pneumoniae*, food sector, transmission, chicken meat, salads, antibiotic resistance, One Health, genomics, surveillance, culture methods

## Abstract

The Klebsiella pneumoniae species complex (KpSC) is a leading cause of multidrug-resistant human infections. To better understand the potential contribution of food as a vehicle of KpSC, we conducted a multicentric study to define an optimal culture method for its recovery from food matrices and to characterize food isolates phenotypically and genotypically. Chicken meat (*n* = 160) and salad (*n* = 145) samples were collected in five European countries and screened for the presence of KpSC using culture-based and *zur*-*khe* intergenic region (ZKIR) quantitative PCR (qPCR) methods. Enrichment using buffered peptone water followed by streaking on Simmons citrate agar with inositol (44°C for 48 h) was defined as the most suitable selective culture method for KpSC recovery. A high prevalence of KpSC was found in chicken meat (60% and 52% by ZKIR qPCR and the culture approach, respectively) and salad (30% and 21%, respectively) samples. Genomic analyses revealed high genetic diversity with the dominance of phylogroups Kp1 (91%) and Kp3 (6%). A total of 82% of isolates presented a natural antimicrobial susceptibility phenotype and genotype, with only four CTX-M-15-producing isolates detected. Notably, identical genotypes were found across samples—same food type and same country (15 cases), different food types and same country (1), and same food type and two countries (1)—suggesting high rates of transmission of KpSC within the food sector. Our study provides a novel isolation strategy for KpSC from food matrices and reinforces the view of food as a potential source of KpSC colonization in humans.

**IMPORTANCE** Bacteria of the Klebsiella pneumoniae species complex (KpSC) are ubiquitous, and K. pneumoniae is a leading cause of antibiotic-resistant infections in humans. Despite the urgent public health threat represented by K. pneumoniae, there is a lack of knowledge of the contribution of food sources to colonization and subsequent infection in humans. This is partly due to the absence of standardized methods for characterizing the presence of KpSC in food matrices. Our multicentric study provides and implements a novel isolation strategy for KpSC from food matrices and shows that KpSC members are highly prevalent in salads and chicken meat, reinforcing the view of food as a potential source of KpSC colonization in humans. Despite the large genetic diversity and the low levels of resistance detected, the occurrence of identical genotypes across samples suggests high rates of transmission of KpSC within the food sector, which need to be further explored to define possible control strategies.

## INTRODUCTION

Klebsiella pneumoniae, a common gut bacterium, is regarded as a critical-priority pathogen (1, 2), given the depletion of therapeutic options to treat multidrug-resistant (MDR) K. pneumoniae infections. Furthermore, hypervirulent community-acquired invasive infections represent a growing problem, especially in Asian countries (3). Concerning reports of the convergence of MDR and hypervirulent phenotypes are increasing (4). The clinical epidemiology of K. pneumoniae is dominated by the clonal spread of important MDR and hypervirulent sublineages, such as clonal group 258 (CG258), CG15, CG147, and CG23 (3).

The ecology and transmission of K. pneumoniae outside the clinical setting is still underexplored; in particular, the reservoir and possible contribution of nonclinical sources in the transmission of prominent clonal groups to humans remain largely undefined. Given the broad ecological distribution of K. pneumoniae in animals and the environment (5, 6) and its capacity to contaminate food (7–9), the contribution of food sources to colonization of humans, and to subsequent infection, is an important question to address. Currently, the detection, isolation, and identification of K. pneumoniae are not well integrated in food microbiological surveillance programs. There is also a lack of standardized tools and procedures for the detection of K. pneumoniae in food. To define the natural ecology and transmission of K. pneumoniae, studies of the presence of K. pneumoniae in food should be designed irrespective of antimicrobial resistance phenotypes.

In order to achieve precise detection and identification of K. pneumoniae, it is important to define the target species. Recent taxonomic updates pointed out the existence of seven phylogroups (phylogroup 1 [Kp1] to Kp7), now defined as distinct taxa, within Klebsiella pneumoniae *sensu lato*. The resulting K. pneumoniae species complex (KpSC) consists of five species, K. pneumoniae *sensu stricto* (Kp1), K. quasipneumoniae (subsp. *quasipneumoniae* [Kp2] and subsp. *similipneumoniae* [Kp4]), K. variicola (subsp. *variicola* [Kp3] and subsp. *tropica* [Kp5]), “*K. quasivariicola*” (Kp6, which remains to be formally defined), and K. africana (Kp7) (10–12). Recently, Barbier et al. developed *zur*-*khe* intergenic region (ZKIR) quantitative PCR (qPCR) (13) for the detection of KpSC in soil and environmental samples, but its application to food matrices was not evaluated. Regarding the isolation of Klebsiella spp., different selective culture methods (7, 14, 15) have been described, but comparisons of their efficiency for Klebsiella isolation from food are lacking.

The aims of this study were (i) to compare the selectivity, productivity, and specificity of three agar media for the detection and isolation of Klebsiella spp., leading to the proposal of a standardized culture protocol for the recovery of Klebsiella spp. in food matrices and comparison of its performance with the ZKIR qPCR and (ii) to evaluate the presence and phenotypic and genomic features of KpSC in two common globally consumed foodstuffs, chicken meat and ready-to-eat salads, through a multicentric study in five European countries.

## RESULTS

### Comparison of culture media.

Productivity (P_R_), selectivity (S_F_), and specificity were calculated separately for each of the media considered. The three media were compliant with ISO 11133:2014 requirements for productivity, as P_R_ was above 0.5 for almost all target strains (see Fig. S1 in the supplemental material). However, two strains had P_R_ of <0.50—K. pneumoniae SB132 in chromatic detection agar and *K. quasipneumoniae* subsp. *quasipneumoniae* SB1124, for which colony growth was only observed on Simmons citrate agar with inositol (SCAI) medium.

We observed that the media did not comply with the selectivity criteria (S_F_, >2) for most of the nontarget strains considered (Table S1); the S_F_ values ranged from −0.3 to 0.5 for Klebsiella ChromoSelect agar and from −0.5 to 0.9 for SCAI medium (chromatic detection agar from Liofilchem was not tested, as it is not selective). We noted that Klebsiella ChromoSelect agar was selective for *Cronobacter* spp. and Citrobacter freundii, showing S_F_ values of 7.6 and 4, respectively. Hence, the media cannot be considered selective for Klebsiella according to the ISO 11133:2014 requirements.

Regarding specificity, the selective media tested showed variable results regarding the morphological features of observed colonies, based on the nontarget strains considered. The selective media, indeed, allowed the growth of some non-Klebsiella species strains, and in few cases, colonies were morphologically similar to the target strains. Overall, because K. pneumoniae colonies were easily distinguishable on SCAI medium and because it was as performant as the others regarding the other criteria, the SCAI medium was selected and used here.

### Definition of an optimized protocol for recovery of KpSC from food samples.

Our initial comparisons of the four different culture protocols (Fig. S2) showed that the most effective method for recovery of KpSC from chicken meat was protocol 1B, which consists of a one-step enrichment in buffered peptone water (BPW). KpSC was recovered from 7 out of 36 (19.4%) samples using this protocol, while only 3 (8.3%) samples were positive using protocol 2 (enrichment in LB broth supplemented with ampicillin) and only 2 (5.6%) using protocol 1C (double enrichment—enrichment in BPW followed by enrichment in LB broth supplemented with ampicillin). No KpSC was isolated using direct plating (protocol 1A).

Further, using BPW enrichment of chicken meat (protocol 1B), a higher recovery of KpSC was observed when streaking a 10-µL loop onto SCAI plates versus spreading 10 µL or 100 µL of bacterial enrichment. Of the 28 chicken meat samples examined using both methods, KpSC was recovered from 24 (85.7%) samples using the 10-µL loop versus 19 (79.2%) samples after spreading 10 µL and 7 (29.2%) samples after spreading 100 µL onto SCAI medium. Thus, protocol 1B combined with streaking was used.

### Evaluation of the SCAI plate incubation temperature.

**FIG 1:**
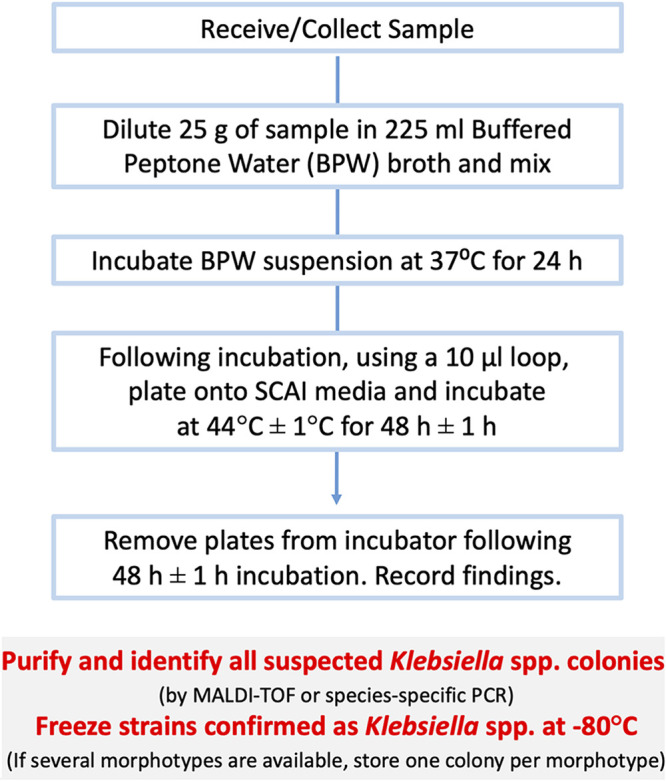
Optimized protocol for the recovery and isolation of Klebsiella spp. from food matrices.

We compared two temperatures (37°C and 44°C) for the incubation of SCAI plates (Fig. S3). Collectively, 111 chicken samples were tested, and 67.7% (64/111) of them were positive for KpSC. KpSC was recovered from 59/111 (53%) samples when SCAI medium was incubated at 37°C, as well as when incubated at 44°C, indicating no difference between incubation temperatures (Table S2). However, when data from each individual institution were analyzed, a slightly higher percentage of positive samples was recorded following incubation of SCAI plates at 44°C at two institutions (Agency for Health and Food Safety [AGES] and National University of Ireland Galway [NUIG]), whereas contrasting results were observed at the third institution (Statens Serum Institute [SSI]) (Table S2). As a result, a decision was made to implement incubation of SCAI plates at 44°C ± 1°C, which was incorporated into our protocol (Fig. 1) (dx.doi.org/10.17504/protocols.io.baxtifnn). All further food samples were tested according to this protocol. Of note, no statistical differences were found in the prevalence of KpSC in free-range and not free-range chicken meat samples (Table S2).

### Comparison of culture and ZKIR qPCR methods for KpSC detection.

Chicken meat and salad leaf samples were tested for the presence of KpSC using both the ZKIR qPCR assay (13) and the optimized culture protocol (Fig. 1). Regarding the chicken meat samples (*n* = 160), the ZKIR qPCR method detected KpSC in 96 (60.0%) of the samples (Table 1). In comparison, when the culture protocol was used, 83 (51.9%, *P = *0.177) of the samples were positive for KpSC (82 K. pneumoniae and 1 *K. variicola* according to matrix-assisted laser desorption ionization–time of flight mass spectrometry [MALDI-TOF MS] results). Regarding salad leaf samples (*n* = 145), 29.7% (43/145) of the samples tested positive for KpSC using the ZKIR qPCR assay, versus 20.7% (30/145; *P = *0.104; 18 K. pneumoniae and 12 *K. variicola* according to MALDI-TOF MS results) using the culture method (Table 1). Considering the ZKIR qPCR assay as a reference, the optimized culture protocol showed a sensitivity (true-positive rate) of 84.2%, a specificity (false-positive rate) of 100%, a positive predictive value of 100%, and a negative predictive value of 86.6%.

**TABLE 1:** Comparison of the recovery rates of K. pneumoniae species complex using the ZKIR qPCR assay versus the optimized culture method

Source	No. of samples	qPCR positive samples (%)	Culture positive samples (%)
Chicken meat	160	96 (60.0%)	83 (51.9%)
Salads	145	43 (29.7%)	30 (20.7%)
Total	305	139 (45.6%)	113 (37.1%)

### Comparison of KpSC prevalence in chicken meat and salad samples.

**FIG 2:**
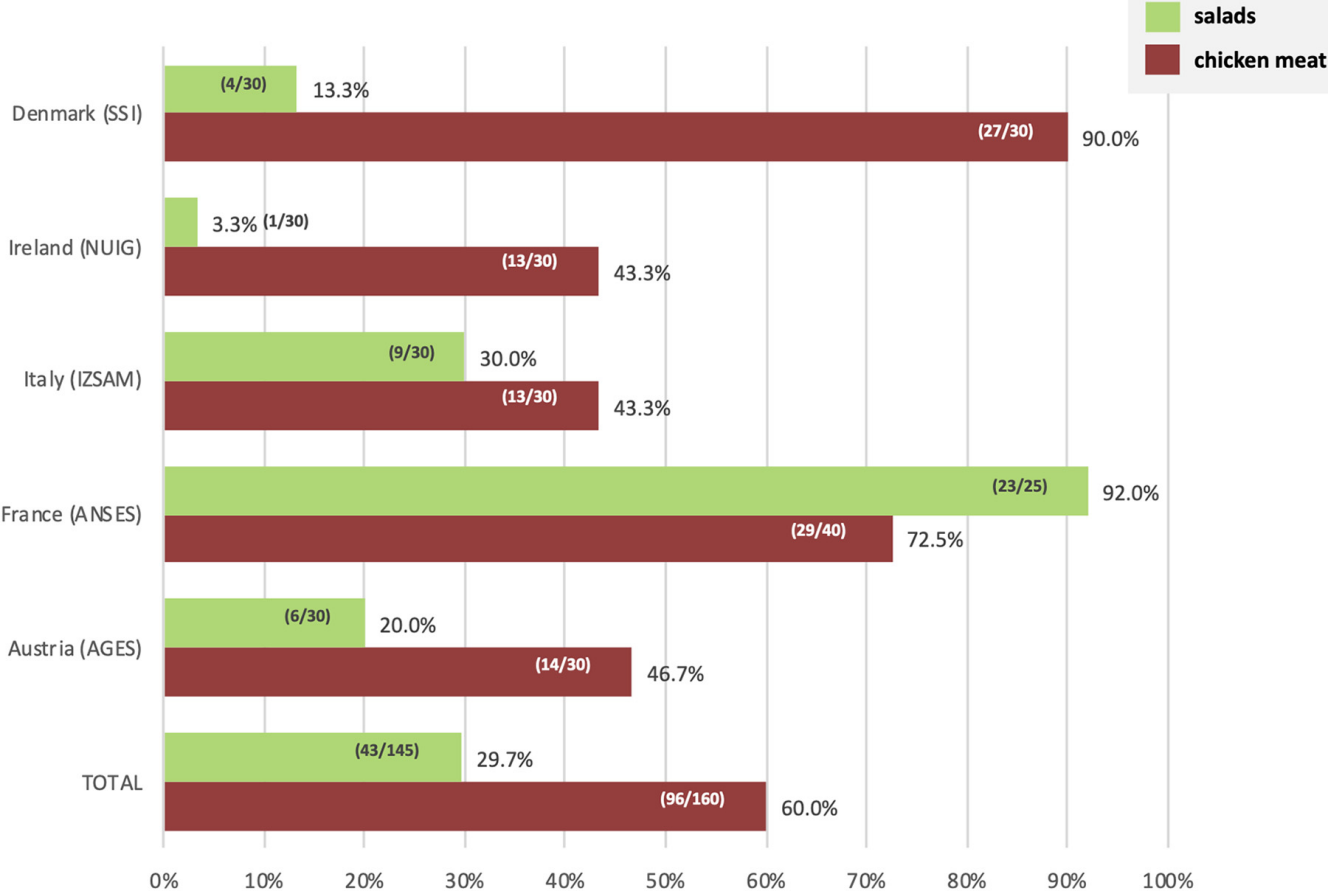
Prevalence of K. pneumoniae species complex by type of sample and by country.

The overall prevalence of KpSC in chicken meat was twice as high as that in salad samples (60.0% versus 29.7%; *P < *0.00001; Table 1). However, prevalence differed among countries (Fig. 2). The prevalence of KpSC in chicken meat samples ranged between 43.3% and 46.6% for three of the countries (Austria, Italy, and Ireland), whereas in France and Denmark the prevalence was above 70% (72.5% and 90.0%, respectively). Regarding salad samples, the prevalence of KpSC ranged from 3.3% in Ireland to 92% in France, with Austria, Italy, and France being the countries where the highest numbers of samples positive for KpSC were detected.

In total, 131 KpSC isolates (92 from chicken meat and 39 from salads) were collected from 113 positive samples using the optimized culture method; in some positive samples, up to 5 different morphotypes were collected (Fig. 2, Table S3).

### Antimicrobial susceptibility.

Antimicrobial susceptibility phenotypes were defined for the 131 KpSC isolates using 17 antimicrobial agents representing 11 antimicrobial classes (Table S4); 82% of the isolates presented a wild-type phenotype, being susceptible to all the antibiotics tested except ampicillin, for which constitutive resistance is a characteristic of all KpSC isolates. The highest rates of resistance (detected in >5% of the isolates) were observed for trimethoprim (12% in chicken meat and 13% in salads), trimethoprim-sulfamethoxazole (8% for both), tetracycline (9% in chicken meat and 5% in salads), and piperacillin-tazobactam (7% in chicken meat; Fig. S4, Table S4). Four isolates (2 from each source) were found to be resistant to extended-spectrum cephalosporins, and the presence of extended-spectrum β-lactamase (ESBL) genes in the genomic sequence was confirmed (Table S4). No isolate was found to be resistant to cefoxitin, ertapenem, or netilmicin. Similar levels of resistance were detected among isolates from salads and from chicken meat.

Differences in antimicrobial resistance occurrence rates were observed among countries (Table S4), but the low number of isolates implies precaution in the analysis. For chicken meat, higher resistance rates were detected in Italy (8 to 50%), Austria (7 to 23%), and Ireland (7 to 14%), whereas for salads it was in Italy (33 to 100%), Austria (20%), and France (4 to 12%) (Fig. S4, Table S4).

Nine (6.9%) multidrug-resistant isolates were identified (Table S5), including the four ESBL producers. MDR isolates were recovered in 7 chicken meat and 2 salad samples in Italy (*n* = 3), Austria (*n* = 2), France (*n* = 2), and Ireland (*n* = 2) (Table S5).

### Phylogenetic and genomic diversity.

Based on genomic data, we observed among the KpSC isolates the predominance of Kp1 (90.8%, 119/131) followed by Kp3 (6.1%, 8/131), Kp2 (2.3%, 3/131), and Kp4 (0.8%, 1/131). The predominance of Kp1 was found in both sample types (98% in chicken meat; 74% in salads); however, there was an association of Kp3 with salad samples (18% in salads versus 1% in chicken meat; *P = *0.001). Kp2 was only found in salads, whereas Kp4 was only detected in chicken meat (Table S6).

**FIG 3:**
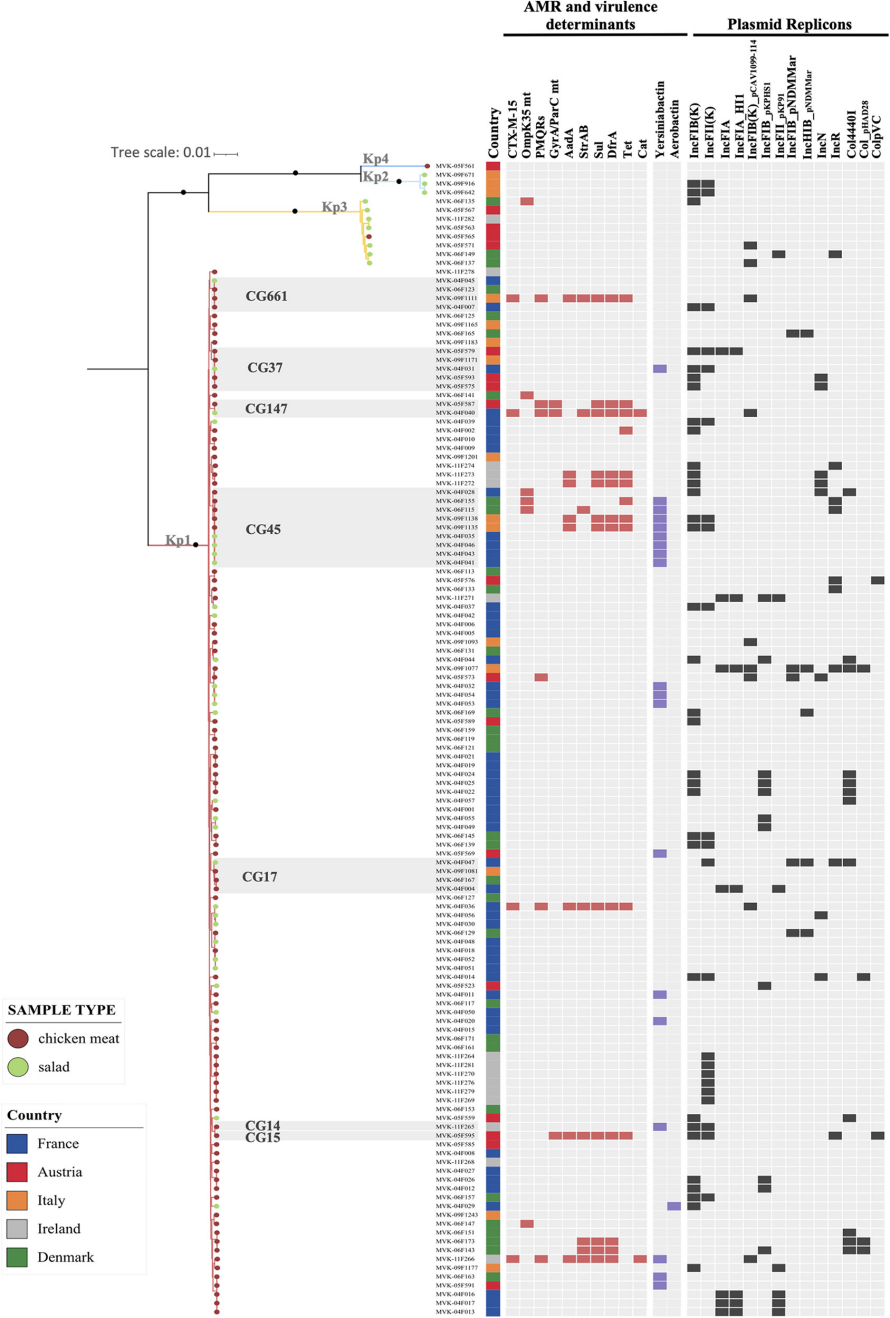
Maximum likelihood phylogeny based on 4,125 concatenated core genes generated using IQ-Tree with the GTR+F+ASC+R5 model. The scale bar indicates the number of nucleotide substitutions per site. Black dots on the main nodes indicate bootstrap values of ≥95%. Tree tips are colored by sample type (see key). The gray boxes delineate high-risk clonal groups (CG), as specified. European countries where the samples were collected are colored as indicated in the legend. The presence of antimicrobial resistance and virulence genes and plasmid replicons is indicated.

Population structure analysis based on multilocus sequence type (MLST) and core genome MLST (cgMLST) genotyping unveiled high genetic diversity among the KpSC isolates, with 86 sequence types (STs) (19 of them defined in this study; Simpson’s index of diversity [SID], 0.989) and 107 cgSTs (SID, 0.995; Fig. 3). High-risk clonal groups (CGs) common in the clinical setting represented 19.8% (26/131) of the isolates (Table S6). Among them, CG45 (*n* = 9), CG37 (*n* = 5), CG17 (*n* = 4), CG661 (*n* = 4), CG147 (*n* = 2), CG14 (*n* = 1), and CG15 (*n* = 1) were found in chicken meat (*n* = 18; 11.3%) and/or salad (*n* = 6, 3.8%) samples. A high diversity of capsular types was also found, with 71 *wzi*-alleles and 58 capsular locus (KL)-types. In contrast, O antigen (O)-type diversity was low, with O1 (25.1%; 33/131) and O2 (20.6%; 27/131) representing almost 50% of the recovered KpSC, followed by O3 (28.2%; 37/131) (Fig. 3).

Defining single strains (designed as genotypes) as isolates differing by no more than 5 cgMLST alleles (out of 629 loci) (16), we observed 15 strains that were isolated at least twice across a total of 35 samples. These were collected from the same food type (chicken meat or salads) at different times in the same country (Table S6, Fig. S5). To improve the confidence inferring the close relationship between these cgMLST-based overlapped genotypes, the single-nucleotide polymorphism (SNP) matrix based on 4,125 core genes obtained from Roary (representing ∼75% of a typical K. pneumoniae genome length against 10% represented by the 629 cgMLST genes) (16) was used and confirmed the close genetic relatedness. For example, in France, the same genotype (ST45/genotype 7; 1 to 4 SNPs) was detected in 3 salad samples from different brands (separated in time by 9 days), whereas in Ireland the same genotype (ST4384/genotype 13; 1 to 8 SNPs) was detected in 6 different chicken meat samples (separated in time by 2 months) from different suppliers (Table S6, Fig. S5). There was one case of the same strain being detected in different countries, as two Kp1 ST290 isolates (genotype 10; 15 SNPs) were detected in chicken meat samples from Denmark (April 2019) and Austria (September 2019) (Fig. S5). We also detected a single strain between chicken meat and salads (ST1537/genotype 9; 23 SNPs) between May and September 2019, respectively, in France (Table S6).

In most isolates (82%; 108/131) only intrinsic *bla*_SHV/OKP/LEN_ genes were detected, consistent with a natural susceptibility genotype (Table S6). Few antibiotic resistance genes were observed across the 131 isolates, with genes of nonprominent clinical significance *sul* and *dfr* (9.2%; 12/131 both), *tet* (8.4%; 11/131), and *aadA* and *strAB* (6.1%; 8/131 both) being the most frequent ones. However, four CTX-M-15-producing isolates were detected in two salad samples from France and two chicken meat samples from Italy and Ireland. The genotype of MDR isolates was compatible with their phenotype (Table S5). No carbapenemase or colistin resistance genes or mutations were detected. In 45.8% (60/131) of the isolates, neither plasmid replicons nor plasmid-encoded heavy metal tolerance genes were detected. In strains where plasmid replicons were detected, the plasmids known to occur naturally in K. pneumoniae, such as IncFIB and IncFII_K_, were the most prevalent (25.2% and 16.8%, respectively). Regarding virulence genes, the majority of KpSC isolates from foodstuffs displayed a virulence score of 0 (Table S6). We found that only 15% (20/131) of strains showed a virulence score different from 0, with the majority of them (14.5%; 19/131) having a virulence score of 1, corresponding to the presence of a yersiniabactin gene cluster. Only one isolate displaying a virulence score of 3, as defined by the presence of aerobactin (*iuc3*), was detected (MVK-04F029-ST4867/KL51) in one salad sample from France.

## DISCUSSION

Despite the renewed interest in Klebsiella pneumoniae species complex (KpSC) epidemiology, boosted by the increasing involvement of K. pneumoniae *sensu stricto* (Kp1) in human infections associated with high levels of antibiotic resistance and/or virulence (1, 3), the contribution of nonclinical sources, such as food products, to the current emergence of KpSC is poorly understood. This is partly due to the lack of standardized protocols for the detection and isolation of K. pneumoniae strains in environmental, food, or animal samples. Different selective culture media have been previously developed for Klebsiella spp. Among the most recognized are MacConkey-inositol-carbenicillin agar (MCIC) (17) and its variation containing adonitol (MCICA) (18), SCAI (14, 19), and brilliant green containing inositol-nitrate-deoxycholate agar (BIND) (20). All of them rely on the ability of Klebsiella spp. to use inositol as a carbon source, combined with the use of secondary selective compounds (e.g., citrate as a carbon source in the case of SCAI; nitrate as a nitrogen source in BIND; carbenicillin and adonitol in MCIC/MCICA, although only Kp1 and Kp2/Kp4 metabolize adonitol) (11). In our study, we decided to evaluate the performance of three culture media for the detection and isolation of Klebsiella spp. in accordance with ISO 11133:2014 and the latest amendments. We included the noncommercial SCAI medium and two commercial culture media, the Klebsiella ChromoSelect selective agar produced by Sigma-Aldrich, which similar to MCIC uses carbenicillin as a selective compound; and chromatic detection agar developed by Liofilchem, which is a chromogenic nonselective medium allowing the identification of Klebsiella spp., Enterobacter spp., and *Serratia* spp. based on the green-blue color of colonies (http://www.liofilchem.net/login/pd/ifu/11611_IFU.pdf). The productivity tests of the three media did not show any critical issues, whereas none of the media tested fulfilled the selectivity and specificity criteria defined by ISO 11133:2014. Nevertheless, the distinction of Klebsiella spp. colonies from other species was achieved in general. This is a critical point, as the routinely used media (e.g., MacConkey or cystine-lactose-electrolyte-deficient [CLED] agar) do not provide easy differentiation of Klebsiella spp. colonies from other species, such as *Citrobacter* spp., *Pantoea* spp., or *Serratia* spp. Here, the performance of the tested culture media showed similar results, and SCAI was selected, due to its low cost and easy in-house preparation. The use of this medium in recent Klebsiella spp. epidemiological studies may also represent an advantage for global and cross-sector comparisons (13, 21–23).

Once SCAI medium was selected, different protocols to isolate Klebsiella spp. from food matrices were evaluated. Multiple conditions, involving preenrichment, enrichment, incubation temperature, and plating, were assessed from chicken meat samples. The optimized culture protocol was then compared with the reference ZKIR qPCR assay in a multicentric design study involving chicken meat and salad samples (13). Overall, KpSC was detected at a higher rate in both chicken meat and salad samples using the ZKIR qPCR method, but even so, the optimized culture protocol demonstrated good sensitivity (84%) and specificity (100%) rates.

Approximately 50% of the food samples tested were KpSC positive, with significantly higher occurrence in chicken meat compared to salads, although disparities in prevalence were observed according to the country analyzed. It is difficult to contextualize our findings in a global scenario since few studies have addressed KpSC prevalence in these type of products without previous antibiotic selection; most of the studies addressing the prevalence and characterization of KpSC from foodstuffs have focused on colistin-resistant or ESBL- or carbapenemase-producing K. pneumoniae (8, 24–26). As a consequence, knowledge of the ecology and natural diversity of K. pneumoniae populations has been very limited. Furthermore, identification of the bacterial species was imprecise in most previous studies, in light of recent taxonomic changes. Our broader approach also captured susceptible strains and led to several important observations. First, the KpSC prevalence in chicken meat was higher than previously described—47% in the United States (Arizona) in 2012 (7), 30% in Turkey in 2007 to 2008 (27), and 14% in China (Shijiazhuang) in 2013 to 2014 (28). Second, despite the high prevalence of KpSC in chicken meat samples, ESBL-producing K. pneumoniae was only detected in 1.3% (2 CTX-M-15) of the samples. These numbers are similar to those reported in other studies that targeted ESBL strains directly by selective approaches (0 to 5%) (8, 26, 28–30). Notably, the presence of ESBL in chicken meat was scarce in K. pneumoniae compared with Escherichia coli (55 to 92%) in recent European reports (26, 30–32).

Third, the highest resistance rates detected in natural food K. pneumoniae populations were to tetracyclines, trimethoprim, and trimethoprim-sulfamethoxazole, reflecting the recent reports of sales of these antimicrobial classes for use in food-producing animals in different European countries (33). Fourth, despite the predominance of Kp1 and the high genetic diversity found among chicken meat isolates, some genotypes overlapped across samples. The relation of these common genotypes (≤5 allele mismatches) with the core-genome alignment obtained from Roary was also explored, and in most of the cases, less than 21 SNPs were identified, a threshold recently proposed for K. pneumoniae ST258 to discriminate hospital clusters (34). Here, we found a maximum of 42 SNPs detected within the same genotype. Some of these multisample genotypes (ST290/genotype 10 recovered in Austria and Denmark and ST45/genotype 15 recovered in Denmark) were previously found circulating among healthy poultry in France in 2015 (C. Rodrigues and M. Haenni, personal communication). The repeated isolation of single strains in unrelated samples across time suggests transmission from the same source/reservoir or transmission within chicken flocks during production. Importantly, MDR ST45/genotype 11 found in Italy has also been found in urinary tract infections in Italian patients in 2018 (22), although with fewer antibiotic resistance genes detected (*sul1*, *dfrA1*, and *aadA1* not present in clinical isolates), suggesting a contribution of food KpSC to human infections (7, 24).

Salads are typically eaten raw, with a high risk of ingestion of live K. pneumoniae bacteria if present. The prevalence of KpSC in ready-to-eat salads was approximately 30%, higher than what was reported in the few comparable studies (6 to 15%) (35–38). ESBL producers were found in 1.4% of the samples (2 CTX-M-15), a lower prevalence than what is described in the literature (15 to 25%) targeting ESBL strains (38–41). As observed for chicken meat samples, the same genotype was observed within the same country in salads from the same or different chain supplier/supermarket, suggesting a common source of the vegetables (e.g., different brands supplied by a common farm) or cross-contamination during their growth and processing (e.g., use of untreated irrigation water, wild-life animals, contamination of fields with manure, human contamination during packaging). Furthermore, one case of genotype overlap was found between salads and chicken meat isolates from samples from France, suggesting local transmission.

A previous study of risk factors of K. pneumoniae carriage in community settings has linked the consumption of raw vegetables and contact with chicken to MDR KpSC carriage in humans (21). These findings together with our results, which are based on a small sampling survey, highlight the possible role of food as a source of K. pneumoniae and call for much broader studies to address the ecology and transmission of this generalist pathogen in the food sector.

In our sampling, sublineages defined as high-risk clonal groups (3) represented 20% of the isolates recovered. One of these, ST45 (in both type of samples) was highly prevalent, as well as ST290 (in chicken meat), and the potential risk they pose should be addressed in future studies. Both STs have already been linked to human infections (22, 42, 43) and human carriage (21, 22) and also to broiler and pig production (23, 44), raising the question of the zoonotic potential of these STs.

### Conclusions.

In conclusion, our study addressed important knowledge gaps relating to K. pneumoniae in food sources. First, we addressed the lack of harmonized culture protocols for the detection and isolation of Klebsiella spp. from food matrices, with the development of an optimized SCAI-based culture protocol, which in the future may be more widely adopted to enable global comparison regarding KpSC prevalence in these types of sources. Second, the high prevalence of KpSC in chicken meat and ready-to-eat salads highlights the possible role of food as a source of human colonization and infection by KpSC. Even though KpSC strains detected in food products remain largely susceptible to antimicrobials, understanding the degree to which food contamination by K. pneumoniae contributes to human infections is an important topic for future research in the One Health perspective.

## MATERIALS AND METHODS

### Comparison of productivity, selectivity, and specificity of agar media.

Three different selective solid media for Klebsiella spp. growth were tested in accordance with ISO 11133:2014 (“Microbiology of Food, Animal Feed and Water—Preparation, Production, Storage and Performance Testing of Culture Media” https://www.iso.org/standard/53610.html). Nutrient agar (Microbiol, Cagliari, Italy) was used as; the nonselective reference medium. For productivity, selectivity, and specificity assays, a set of 58 strains (50 from Institut Pasteur and 8 from Istituto Zooprofilattico Sperimentale dell’Abruzzo e del Molise Giuseppe Caporale [IZSAM]) were used—51 reference strains of Klebsiella spp. and closely related species (*Raoultella* spp.), which included representatives of the six main KpSC phylogroups, and 7 nontarget strains (Table S7). For productivity (P_R_), the three following media were tested: Simmons citrate agar with inositol (SCAI) (14), Klebsiella ChromoSelect selective agar produced by Sigma-Aldrich (Missouri, USA), and chromatic detection agar developed by Liofilchem (Roseto degli Abruzzi, Italy). The SCAI medium is not available commercially and was prepared in-house following previous protocols (14, 19) (https://www.protocols.io/view/isolation-of-klebsiella-strains-from-human-or-anim-662hhge/materials). P_R_ was calculated using 50 reference strains belonging to Klebsiella spp. and closely related species (*Raoultella* spp.) (Table S7). After incubation at 37°C for 24 h, broth cultures in brain heart infusion (BHI) (Biolife, Milan, Italy) of each strain were diluted up to about 100 CFU/mL and spread on media, with the dilutions being confirmed by plate count on nutrient agar. For all the strains and the three media considered, the P_R_ was evaluated at the same time and from the same inoculum dilution of each target strain considered. After incubation at 37°C for 48 h, colonies compatible with the Klebsiella phenotype were enumerated. P_R_ is calculated as the ratio between the number of colonies grown on the selective agar medium and the number of colonies grown on the nonselective medium, and according to ISO 11133:2014, P_R_ values should be ≥0.50 to consider the medium productivity acceptable.

For selectivity (S_F_), 7 nontarget strains (*Cronobacter* spp., Citrobacter koseri, Citrobacter freundii, Serratia marcescens, Serratia liquefaciens, Serratia rubidaea, and Pantoea agglomerans) were tested on the SCAI and Klebsiella ChromoSelect selective agar (Table S7). After incubation at 37°C for 24 h in BHI, broth cultures were 10-fold diluted serially, and each dilution was spread onto plates. After incubation at 37°C for 48 h, colony growth was observed. S_F_ is defined as the difference between the highest dilution showing growth on the nonselective reference medium (log_10_) and the highest dilution showing comparable growth on the selective test medium (log_10_). In accordance with ISO 11133:2014, S_F_ values of nontarget strains should be at least equal to 2 log_10_ growth differences, which reflects the ability of the medium to partially or totally inhibit their growth.

For specificity, the 7 nontarget strains and two control strains (1 K. pneumoniae and 1 Raoultella ornithinolytica) were tested on the three media (Table S7). Broth cultures were prepared in buffered peptone water (BPW) (Biolife, Milan, Italy), incubated at 37°C for 24 h, and spread onto each medium considered. After incubation at 37°C for 48 h of each medium, plates were checked by two observers for the presence of typical and suspected colonies, recording if the characteristics of the nontarget colonies could be similar to the target ones.

### Initial evaluation of protocols for recovery of Klebsiella pneumoniae from food.

An initial assessment of four protocols for recovery of KpSC from chicken meat was carried out. In total, 36 chicken meat samples (12 free-range [7 skin-on and 5 skin-off] and 24 non-free range [12 skin-on and 12 skin-off] samples) were collected together with the following metadata: manufacturer, country of origin, and batch number. Each chicken meat sample was cut into small slivers to a final weight of 50 g. This 50-g sample was divided into two portions of 25 g and processed as follows and as illustrated in Fig. S2.

**Initial suspension 1.** The first 25-g portion was diluted in 225 mL (1:10 dilution) of BPW, and the sample was homogenized using a stomacher/blender. Prior to incubation of initial suspension 1, 100 µL of the suspension was cultured directly on SCAI medium using a sterile spreader or sterile loop and incubated at 37°C ± 1°C for 48 h ± 1 h (Fig. S2 [1A, detection without enrichment]). The remainder of initial suspension 1 was incubated at 37°C ± 1°C for 24 h ± 1 h. Following 24 h of incubation of initial suspension 1, 100 µL of the suspension was streaked on SCAI medium and incubated at 37°C ± 1°C for 48 h ± 1 h (Fig. S2 [1B, detection with BPW enrichment only]). Of the remaining incubated suspension 1, 1 mL was inoculated into 9 mL of lysogeny broth (LB) with ampicillin (final concentration, 0.01 mg/mL) and incubated at 37°C ± 1°C for 24 h ± 1 h. Following 24 h of incubation, 100 µL of the suspension was streaked on SCAI medium and incubated at 37°C ± 1°C for 48 h ± 1 h (Fig. S2 [1C, detection with double enrichment]).

**Initial suspension 2.** The second 25-g portion was diluted in 225 mL (1:10 dilution) of LB broth supplemented with ampicillin (final concentration 0.01 mg/mL), and the sample was homogenized using a stomacher/blender and incubated at 37°C ± 1°C for 24 h ± 1 h. Following 24 h of incubation, 100 µL of the suspension was cultured on SCAI medium and incubated at 37°C ± 1°C for 48 h ± 1 h (Fig. S2 [2, detection with LB + ampicillin enrichment only]).

Typically, Klebsiella spp. appear yellow on SCAI medium (14). At least five suspected Klebsiella colonies were collected from each SCAI plate and subcultured onto SCAI medium or a nonselective agar for identification using MALDI-TOF MS (Bruker Daltonics, Bremen, Germany). Currently, databases only allow for the identification of K. pneumoniae or *K. variicola*. Based on the literature and in our test strain set, Kp1, Kp2, and Kp4 strains are identified as K. pneumoniae, whereas Kp3, Kp5, and Kp6 are currently identified as *K. variicola* (45, 46).

For 28/36 chicken meat samples, the impact on KpSC recovery of culturing using a 10-µL loop versus the spreading of 10 µL and 100 µL of enrichments onto SCAI medium was also compared.

### Evaluation of the optimized protocol and definition of an optimal temperature of incubation.

Following this initial assessment process, the optimized protocol as outlined below and as illustrated in Fig. S3 was evaluated for recovery of KpSC from chicken meat samples using a total of 111 samples of chicken meat (51 free-range [25 skin-on and 26 skin-off] and 60 non-free range [30 skin-on and 30 skin-off]).

Each sample was cut into small slivers to a final weight of 25 g. This was added to 225 mL of BPW broth (1:10 dilution), and the sample was mixed using a stomacher or blender. The suspension was then incubated at 37°C ± 1°C for 24 h ± 1 h. Following incubation, the suspension was cultured for single colonies on SCAI using a 10-µL loop and incubated at two different temperatures, 37°C ± 1°C and 44°C ± 1°C, for 48 h ± 1 h (Fig. S3) in order to establish an optimal temperature of incubation of SCAI medium for recovery of K. pneumoniae from food matrices.

### Comparison of the optimized SCAI culture protocol and ZKIR qPCR.

A comparison of the optimized SCAI culture protocol and the ZKIR qPCR for the detection and isolation of KpSC in food matrices was carried out in 5 European institutions, the Austrian Agency for Health and Food Safety (AGES), French Agency for Food, Environmental and Occupational Health & Safety (ANSES), National University of Ireland Galway (NUIG), Istituto Zooprofilattico Sperimentale dell’Abruzzo e del Molise Giuseppe Caporale (IZSAM), and the Statens Serum Institute (SSI, Denmark).

Each institution was asked to collect and test, where possible, an additional 30 samples of chicken meat, as well as 30 prepackaged, prewashed salad leaf samples (Table S2). In total, 160 chicken meat samples and 145 salad samples, collected between December 2018 and September 2019 in different supermarkets and representing a variety of suppliers (average of 9 suppliers per country) were tested using the updated optimized protocol (dx.doi.org/10.17504/protocols.io.baxtifnn) for recovery of KpSC from food matrices (Fig. 1). In order to avoid external contamination during the laboratory processing, all samples were analyzed in accordance with laboratory practice as reported in ISO 7218:2007, “Microbiology of Food and Animal Feeding Stuffs—General Requirements and Guidance for Microbiological Examination.”

The ZKIR qPCR was used to detect the presence of KpSC in food samples (13). After enrichment, DNA was prepared for ZKIR qPCR. For 500 μL of the enrichment broth was centrifuged (5 min at 5,800 × *g*), washed with sterile water, and resuspended in 500 μL of sterile water before boiling for 10 min. qPCR conditions were as previously described (13) (dx.doi.org/10.17504/protocols.io.7n6hmhe).

### Antimicrobial susceptibility testing.

Antimicrobial susceptibility testing was performed on 131 KpSC isolates using either disk diffusion or broth microdilution (GN2F panels [Sensititre; Thermo Fischer Scientific]) methods. Clinical breakpoints were used for interpretation according to EUCAST guidelines (https://www.eucast.org/fileadmin/src/media/PDFs/EUCAST_files/Breakpoint_tables/v_9.0_Breakpoint_Tables.pdf), except for kanamycin and streptomycin (47). Escherichia coli ATCC 25922 was used as the control strain. The panel of antimicrobials tested included amoxicillin-clavulanic acid (20/10 µg), piperacillin-tazobactam (both using disks of 30/6 µg or GN2F plates), cefpodoxime (10 µg, GN2F) or cefotaxime (5 µg), cefoxitin (30 µg, GN2F), aztreonam (30 µg), ertapenem (10 µg), amikacin (30 µg, GN2F), gentamicin (10 µg, GN2F), netilmicin (10 µg), tobramycin (10 µg, GN2F), kanamycin (10 µg), streptomycin (30 µg), ciprofloxacin (5 µg, GN2F) or ofloxacin (5 µg), nalidixic acid (30 µg), trimethoprim (5 µg), trimethoprim-sulfamethoxazole (23.75 + 1.25 µg, GN2F), and tetracycline (30 µg). The presence of multidrug-resistant isolates, which are defined as those resistant to at least one agent in three different classes of antimicrobials (48), was analyzed.

### Whole-genome sequencing and comparative genomic analyses.

Whole-genome sequencing was performed on the 131 K. pneumoniae isolates. Genomic DNA libraries were prepared using the Nextera XT library prep kit (Illumina, San Diego, USA) following the manufacturer’s protocol. Illumina sequencing was performed at the five partners—AGES and SSI using MiSeq (2 × 250-bp paired-end sequencing; *n* = 18 and *n* = 31 isolates, respectively), ANSES and NUIG using NovaSeq 6000 (2 × 150-bp paired-end sequencing; *n* = 52 and *n* = 15, respectively), and IZSAM using NextSeq 500/550 (2 × 150-bp paired-end sequencing; *n* = 15). Genomic assemblies were obtained using SPAdes v3.9 (49) or Velvet v1.2.10 (50) and were annotated using Prokka v1.12 (51).

Multilocus sequence typing (MLST) (52), core-genome MLST (cgMLST), and cgLIN codes (16) were determined using the BIGSdb-Kp database (https://bigsdb.pasteur.fr/klebsiella/). This web tool and Kleborate (53) (https://github.com/katholt/Kleborate) were used to look for antimicrobial resistance, virulence, and heavy metal tolerance genes and to characterize the capsular synthesis gene cluster. Plasmid replicons were searched using PlasmidFinder (54) (https://cge.cbs.dtu.dk/services/PlasmidFinder/).

For phylogenetic analyses, a core-genome alignment based on the concatenation of 4,125 core genes was obtained with Roary v3.12 (55) using a blastP identity cutoff of 80% and core genes defined as those being present in more than 90% of the isolates. Recombination events were removed using Gubbins v2.2.0 (56), generating a recombination-free alignment comprising 486,290 single-nucleotide variants (SNVs). This recombination-free alignment was used to construct a maximum likelihood phylogenetic tree using IQ-TREE v1.6.11 (model GTR+F+ASC+R5).

Chi square or Fisher exact tests were used to compare the prevalence of KpSC in the different sources and to check the association of the different categorical variables (*P* values of <0.05 were considered statistically significant).

### Data availability.

The detailed optimized SCAI culture protocol for the detection and isolation of KpSC in food matrices was made publicly accessible to the scientific community in January 2020 through the protocols.io platform (dx.doi.org/10.17504/protocols.io.baxtifnn). The genomic sequences generated in this study were submitted to the European Nucleotide Archive and are accessible under the BioProject number PRJEB34643 and are also publicly available in BIGSdb through project ID 37 “MedVetKlebs_multicentric study.”

## References

[1] CDC. 2013. Antibiotic resistance threats in the United States. https://www.cdc.gov/drugresistance/pdf/ar-threats-2013-508.pdf. 25162160

[2] WHO. 2014. Antimicrobial resistance: global report on surveillance . https://apps.who.int/iris/bitstream/handle/10665/112642/9789241564748_eng.pdf?sequence=1&isAllowed=y.

[3] Wyres KL, Lam MMC, Holt KE. 2020. Population genomics of *Klebsiella pneumoniae*. Nat Rev Microbiol 18:344–359. doi:10.1038/s41579-019-0315-1. 32055025

[4] Wyres KL, Wick RR, Judd LM, Froumine R, Tokolyi A, Gorrie CL, Lam MMC, Duchêne S, Jenney A, Holt KE. 2019. Distinct evolutionary dynamics of horizontal gene transfer in drug resistant and virulent clones of *Klebsiella pneumoniae*. PLoS Genet 15:e1008114. doi:10.1371/journal.pgen.1008114. 30986243 PMC6483277

[5] Brisse S, Grimont F, Grimont PAD. 2006. The genus *Klebsiella*. *In* Dworkin M, Falkow S, Rosenberg E, Schleifer K-H, Stackebrandt E, (eds), The prokaryotes A handbook on the Biology of Bacteria 3rd edition. https://link.springer.com/content/pdf/10.1007%2F0-387-30746-X_8.pdf.

[6] Martin RM, Cao J, Brisse S, Passet V, Wu W, Zhao L, Malani PN, Rao K, Bachman MA. 2016. Molecular epidemiology of colonizing and infecting isolates of *Klebsiella pneumoniae*. mSphere 1:e00261-16. doi:10.1128/mSphere.00261-16. 27777984 PMC5071533

[7] Davis GS, Waits K, Nordstrom L, Weaver B, Aziz M, Gauld L, Grande H, Bigler R, Horwinski J, Porter S, Stegger M, Johnson JR, Liu CM, Price LB. 2015. Intermingled *Klebsiella pneumoniae* populations between retail meats and human urinary tract infections. Clin Infect Dis 61:892–899. doi:10.1093/cid/civ428. 26206847 PMC4551003

[8] Ludden C, Moradigaravand D, Jamrozy D, Gouliouris T, Blane B, Naydenova P, Hernandez-Garcia J, Wood P, Hadjirin N, Radakovic M, Crawley C, Brown NM, Holmes M, Parkhill J, Peacock SJ. 2020. A One Health study of the genetic relatedness of *Klebsiella pneumoniae* and their mobile elements in the east of England. Clin Infect Dis 70:219–226. doi:10.1093/cid/ciz174. 30840764 PMC6938978

[9] Zadoks RN, Griffiths HM, Munoz MA, Ahlstrom C, Bennett GJ, Thomas E, Schukken YH. 2011. Sources of *Klebsiella* and *Raoultella* species on dairy farms: be careful where you walk. J Dairy Sci 94:1045–1051. doi:10.3168/jds.2010-3603. 21257074

[10] Brisse S, Passet V, Grimont PAD. 2014. Description of *Klebsiella quasipneumoniae* sp. nov., isolated from human infections, with two subspecies *Klebsiella quasipneumoniae* subsp. *quasipneumoniae* subsp. nov. and *Klebsiella quasipneumoniae* subsp. *similipneumoniae* subsp. nov. and demonstration that Klebsiella singaporensis is a junior heterotypic synonym of Klebsiella variicola. Int J Syst Evol Microbiol 64:3146–3152. doi:10.1099/ijs.0.062737-0. 24958762

[11] Rodrigues C, Passet V, Rakotondrasoa A, Diallo TA, Criscuolo A, Brisse S. 2019. Description of *Klebsiella africanensis* sp. nov., *Klebsiella variicola* subsp. *tropicalensis* subsp. nov. and *Klebsiella variicola* subsp. *variicola* subsp. nov. Res Microbiol 170:165–170. doi:10.1016/j.resmic.2019.02.003. 30817987

[12] Long SW, Linson SE, Saavedra MO, Cantu C, Davis JJ, Brettin T, Olsen RJ. 2017. Whole-genome sequencing of a human clinical isolate of the novel species *Klebsiella quasivariicola* sp. nov. Genome Announc 5:e01057-17. doi:10.1128/genomeA.01057-17. 29051239 PMC5646392

[13] Barbier E, Rodrigues C, Depret G, Passet V, Gal L, Piveteau P, Brisse S. 2020. The ZKIR assay, a real-time PCR method for the detection of *Klebsiella pneumoniae* and closely related species in environmental samples. Appl Environ Microbiol 86:e02711-19. doi:10.1128/AEM.02711-19. 32005732 PMC7082575

[14] Van Kregten E, Westerdaal NAC, Willers JMN. 1984. New, simple medium for selective recovery of *Klebsiella pneumoniae* and *Klebsiella oxytoca* from human feces. J Clin Microbiol 20:936–941. doi:10.1128/jcm.20.5.936-941.1984. 6392324 PMC271478

[15] Filius PMG, van Netten D, Roovers PJE, Vulto AG, Gyssens IC, Verbrugh HA, Endtz HP. 2003. Comparative evaluation of three chromogenic agars for detection and rapid identification of aerobic Gram-negative bacteria in the normal intestinal microflora. Clin Microbiol Infect 9:912–918. doi:10.1046/j.1469-0691.2003.00667.x. 14616678

[16] Hennart M, Guglielmini J, Maiden MCJ, Jolley KA, Criscuolo A, Brisse S. 2021. A dual barcoding approach to bacterial strain nomenclature: genomic taxonomy of *Klebsiella pneumoniae* strains. bioRxiv 2021.07.26.453808. 10.1093/molbev/msac135 PMC9254007 35700230

[17] Bagley S, Seidler R. 1978. Primary *Klebsiella* identification with MacConkey-inositol-carbenicillin agar. Appl Environ Microbiol 36:536–538. doi:10.1128/aem.36.3.536-538.1978. 365108 PMC243082

[18] Gao H, Gao Q-L, Zhang X, Guan C, Luo M-H, Zhang H-B, Liu P, Zhang H-Y, Li J. 2010. Improved medium for detection of *Klebsiella* in powdered milk. J Food Saf 30:12–23. doi:10.1111/j.1745-4565.2009.00186.x.

[19] Passet V, Brisse S. 2015. Association of tellurite resistance with hypervirulent clonal groups of *Klebsiella pneumoniae*. J Clin Microbiol 53:1380–1382. doi:10.1128/JCM.03053-14. 25631812 PMC4365200

[20] Ohtomo R, Saito M. 2003. A new selective medium for detection of *Klebsiella* from dairy environments. Microb Environ 18:138–144. doi:10.1264/jsme2.18.138.

[21] Huynh B-T, Passet V, Rakotondrasoa A, Diallo T, Kerleguer A, Hennart M, De Lauzanne A, Herindrainy P, Seck A, Bercion R, Borand L, Pardos de la Gandara M, Delarocque-Astagneau E, Guillemot D, Vray M, Garin B, Collard J-M, Rodrigues C, Brisse S. 2020. *Klebsiella pneumoniae* carriage in low-income countries: antimicrobial resistance, genomic diversity and risk factors. Gut Microbes 11:1287–1299. doi:10.1080/19490976.2020.1748257. 32404021 PMC7527070

[22] Thorpe H, Booton R, Kallonen T, Gibbon MJ, Couto N, Passet V, Fernandez JSL, Rodrigues C, Matthews L, Mitchell S, Reeve R, David S, Merla C, Corbella M, Ferrari C, Comandatore F, Marone P, Brisse S, Sassera D, Corander J, Feil EJ. 2021. One health or three? Transmission modelling of *Klebsiella* isolates reveals ecological barriers to transmission between humans, animals and the environment. bioRxiv. doi:10.1101/2021.08.05.455249.

[23] Franklin-Alming FV, Kaspersen H, Hetland MAK, Bakksjø R-J, Nesse LL, Leangapichart T, Löhr IH, Telke AA, Sunde M. 2021. Exploring *Klebsiella pneumoniae* in healthy poultry reveals high genetic diversity, good biofilm-forming abilities and higher prevalence in turkeys than broilers. Front Microbiol 12:725414. doi:10.3389/fmicb.2021.725414. 34557173 PMC8453068

[24] Davis GS, Price LB. 2016. Recent research examining links among *Klebsiella pneumoniae* from food, food animals, and human extraintestinal infections. Curr Environ Health Rep 3:128–135. doi:10.1007/s40572-016-0089-9. 27022987

[25] Chaalal N, Touati A, Bakour S, Aissa MA, Sotto A, Lavigne J-P, Pantel A. 2021. Spread of OXA-48 and NDM-1-producing *Klebsiella pneumoniae* ST48 and ST101 in chicken meat in western Algeria. Microb Drug Resist 27:492–500. doi:10.1089/mdr.2019.0419. 32208064

[26] Huizinga P, Kluytmans-van den Bergh M, Rossen JW, Willemsen I, Verhulst C, Savelkoul PHM, Friedrich AW, García-Cobos S, Kluytmans J. 2019. Decreasing prevalence of contamination with extended-spectrum beta-lactamase-producing *Enterobacteriaceae* (ESBL-E) in retail chicken meat in the Netherlands. PLoS One 14:e0226828. doi:10.1371/journal.pone.0226828. 31891609 PMC6938319

[27] Gundogan N, Citak S, Yalcin E. 2011. Virulence properties of extended spectrum β-lactamase-producing *Klebsiella* species in meat samples. J Food Prot 74:559–564. doi:10.4315/0362-028X.JFP-10-315. 21477469

[28] Guo Y, Zhou H, Qin L, Pang Z, Qin T, Ren H, Pan Z, Zhou J. 2016. Frequency, antimicrobial resistance and genetic diversity of *Klebsiella pneumoniae* in food samples. PLoS One 11:e0153561. doi:10.1371/journal.pone.0153561. 27078494 PMC4831839

[29] Cardozo MV, Liakopoulos A, Brouwer M, Kant A, Pizauro LJL, Borzi MM, Mevius D, de Ávila FA. 2021. Occurrence and molecular characteristics of extended-spectrum beta-lactamase-producing *Enterobacterales* recovered from chicken, chicken meat, and human infections in Sao Paulo state, Brazil. Front Microbiol 12:628738. doi:10.3389/fmicb.2021.628738. 34239503 PMC8259509

[30] Díaz-Jiménez D, García-Meniño I, Fernández J, García V, Mora A. 2020. Chicken and turkey meat: consumer exposure to multidrug-resistant *Enterobacteriaceae* including *mcr*-carriers, uropathogenic *E. coli* and high-risk lineages such as ST131. Int J Food Microbiol 331:108750. doi:10.1016/j.ijfoodmicro.2020.108750. 32559710

[31] Day MJ, Hopkins KL, Wareham DW, Toleman MA, Elviss N, Randall L, Teale C, Cleary P, Wiuff C, Doumith M, Ellington MJ, Woodford N, Livermore DM. 2019. Extended-spectrum β-lactamase-producing *Escherichia coli* in human-derived and foodchain-derived samples from England, Wales, and Scotland: an epidemiological surveillance and typing study. Lancet Infect Dis 19:1325–1335. doi:10.1016/S1473-3099(19)30273-7. 31653524

[32] Casella T, Nogueira MCL, Saras E, Haenni M, Madec JY. 2017. High prevalence of ESBLs in retail chicken meat despite reduced use of antimicrobials in chicken production, France. Int J Food Microbiol 257:271–275. doi:10.1016/j.ijfoodmicro.2017.07.005. 28728058

[33] European Medicines Agency. 2020. Tenth ESVAC report: sales of veterinary antimicrobial agents in 31 European countries in 2018. EMA, Amsterdam, Netherlands.

[34] David S, Reuter S, Harris SR, Glasner C, Feltwell T, Argimon S, Abudahab K, Goater R, Giani T, Errico G, Aspbury M, Sjunnebo S, Feil EJ, Rossolini GM, Aanensen DM, Grundmann H, ESGEM Study Group. 2019. Epidemic of carbapenem-resistant *Klebsiella pneumoniae* in Europe is driven by nosocomial spread. Nat Microbiol 4:1919–1929. doi:10.1038/s41564-019-0492-8. 31358985 PMC7244338

[35] Harada T, Taguchi M, Kawahara R, Kanki M, Kawatsu K. 2018. Prevalence and antimicrobial susceptibility of bacterial pathogens in ready-to-eat foods retailed in Osaka Prefecture, Japan. J Food Prot 81:1450–1458. doi:10.4315/0362-028X.JFP-18-035. 30080122

[36] Falomir MP, Rico H, Gozalbo D. 2013. *Enterobacter* and *Klebsiella* species isolated from fresh vegetables marketed in Valencia (Spain) and their clinically relevant resistances to chemotherapeutic agents. Foodborne Pathog Dis 10:1002–1007. doi:10.1089/fpd.2013.1552. 23980710

[37] Boehme S, Werner G, Klare I, Reissbrodt R, Witte W. 2004. Occurrence of antibiotic-resistant enterobacteria in agricultural foodstuffs. Mol Nutr Food Res 48:522–531. doi:10.1002/mnfr.200400030. 15538714

[38] Giri S, Kudva V, Shetty K, Shetty V. 2021. Prevalence and characterization of extended-spectrum β-lactamase-producing antibiotic-resistant *Escherichia coli* and *Klebsiella pneumoniae* in ready-to-eat street foods. Antibiotics 10:850. doi:10.3390/antibiotics10070850. 34356771 PMC8300707

[39] Zurfluh K, Nüesch-Inderbinen M, Morach M, Berner AZ, Hächler H, Stephan R. 2015. Extended-spectrum-β-lactamase-producing Enterobacteriaceae isolated from vegetables imported from the Dominican Republic, India, Thailand, and Vietnam. Appl Environ Microbiol 81:3115–3120. doi:10.1128/AEM.00258-15. 25724954 PMC4393435

[40] Bhutani N, Muraleedharan C, Talreja D, Rana SW, Walia S, Kumar A, Walia SK. 2015. Occurrence of multidrug resistant extended spectrum beta-lactamase-producing bacteria on iceberg lettuce retailed for human consumption. Biomed Res Int 2015:547547. doi:10.1155/2015/547547. 26064922 PMC4433657

[41] Zekar FM, Granier SA, Touati A, Millemann Y. 2020. Occurrence of third-generation cephalosporins-resistant *Klebsiella pneumoniae* in fresh fruits and vegetables purchased at markets in Algeria. Microb Drug Resist 26:353–359. doi:10.1089/mdr.2019.0249. 31603740

[42] Mendes AC, Novais Â, Campos J, Rodrigues C, Santos C, Antunes P, Ramos H, Peixe L. 2018. *mcr*-1 in carbapenemase-producing *Klebsiella pneumoniae* in hospitalized patients, Portugal, 2016–2017. 24:2016–2017. Emerg Infect Dis doi:10.3201/eid2404.171787. PMC5875258 29553327

[43] Safavi M, Bostanshirin N, Hajikhani B, Yaslianifard S, van Belkum A, Goudarzi M, Hashemi A, Darban-Sarokhalil D, Dadashi M. 2020. Global genotype distribution of human clinical isolates of New Delhi metallo-β-lactamase-producing *Klebsiella pneumoniae*: a systematic review. J Glob Antimicrob Resist 23:420–429. doi:10.1016/j.jgar.2020.10.016. 33157280

[44] Leangapichart T, Lunha K, Jiwakanon J, Angkititrakul S, Järhult JD, Magnusson U, Sunde M. 2021. Characterization of *Klebsiella pneumoniae* complex isolates from pigs and humans in farms in Thailand: population genomic structure, antibiotic resistance and virulence genes. J Antimicrob Chemother 76:2012–2016. doi:10.1093/jac/dkab118. 33829268 PMC8283727

[45] Rodrigues C, Passet V, Rakotondrasoa A, Brisse S. 2018. Identification of *Klebsiella pneumoniae*, *Klebsiella quasipneumoniae, Klebsiella variicola* and related phylogroups by MALDI-TOF mass spectrometry. Front Microbiol 9:3000. doi:10.3389/fmicb.2018.03000. 30581423 PMC6294014

[46] Long SW, Linson SE, Ojeda Saavedra M, Cantu C, Davis JJ, Brettin T, Olsen RJ. 2017. Whole-genome sequencing of human clinical *Klebsiella pneumoniae* isolates reveals misidentification and misunderstandings of *Klebsiella pneumoniae*, *Klebsiella variicola*, and *Klebsiella quasipneumoniae*. mSphere 2:e00290-17. doi:10.1128/mSphereDirect.00290-17. 28776045 PMC5541162

[47] Clinical and Laboratory Standards Institute (CLSI). 2017. Performance standards for antimicrobial susceptibility testing, 27th ed. CLSI supplement M100. CLSI, Wayne, PA.

[48] Magiorakos A-P, Srinivasan A, Carey RB, Carmeli Y, Falagas ME, Giske CG, Harbarth S, Hindler JF, Kahlmeter G, Olsson-Liljequist B, Paterson DL, Rice LB, Stelling J, Struelens MJ, Vatopoulos A, Weber JT, Monnet DL. 2012. Multidrug-resistant, extensively drug-resistant and pandrug-resistant bacteria: an international expert proposal for interim standard definitions for acquired resistance. Clin Microbiol Infect 18:268–281. doi:10.1111/j.1469-0691.2011.03570.x. 21793988

[49] Prjibelski A, Antipov D, Meleshko D, Lapidus A, Korobeynikov A. 2020. Using SPAdes *de novo* assembler. Curr Protoc Bioinforma 70:e102. doi:10.1002/cpbi.102. 32559359

[50] Zerbino DR, Birney E. 2008. Velvet: algorithms for de novo short read assembly using de Bruijn graphs. Genome Res 18:821–829. doi:10.1101/gr.074492.107. 18349386 PMC2336801

[51] Seemann T. 2014. Prokka: rapid prokaryotic genome annotation. Bioinformatics 30:2068–2069. doi:10.1093/bioinformatics/btu153. 24642063

[52] Diancourt L, Passet V, Verhoef J, Grimont PAD, Brisse S. 2005. Multilocus sequence typing of *Klebsiella pneumoniae* nosocomial isolates. J Clin Microbiol 43:4178–4182. doi:10.1128/JCM.43.8.4178-4182.2005. 16081970 PMC1233940

[53] Lam MMC, Wick RR, Watts SC, Cerdeira LT, Wyres KL, Holt KE. 2021. A genomic surveillance framework and genotyping tool for *Klebsiella pneumoniae* and its related species complex. Nat Commun 12:4188. doi:10.1038/s41467-021-24448-3. 34234121 PMC8263825

[54] Carattoli A, Zankari E, García-Fernández A, Voldby Larsen M, Lund O, Villa L, Møller Aarestrup F, Hasman H. 2014. *In silico* detection and typing of plasmids using PlasmidFinder and plasmid multilocus sequence typing. Antimicrob Agents Chemother 58:3895–3903. doi:10.1128/AAC.02412-14. 24777092 PMC4068535

[55] Page AJ, Cummins CA, Hunt M, Wong VK, Reuter S, Holden MTG, Fookes M, Falush D, Keane JA, Parkhill J. 2015. Roary: rapid large-scale prokaryote pan genome analysis. Bioinformatics 31:3691–3693. doi:10.1093/bioinformatics/btv421. 26198102 PMC4817141

[56] Croucher NJ, Page AJ, Connor TR, Delaney AJ, Keane JA, Bentley SD, Parkhill J, Harris SR. 2015. Rapid phylogenetic analysis of large samples of recombinant bacterial whole genome sequences using Gubbins. Nucleic Acids Res 43:e15. doi:10.1093/nar/gku1196. 25414349 PMC4330336

