# Supplementary material for: High Prevalence of Klebsiella pneumoniae in European Food Products: a Multicentric Study Comparing Culture and Molecular Detection Methods
Source: Microbiol Spectr. 2022 Feb 23;10(1):e02376-21. doi: 10.1128/spectrum.02376-21 (PMC8865463; doi:10.1128/spectrum.02376-21)

Supplementary Data

High prevalence of *Klebsiella pneumoniae* in European food products: a multicentric study comparing culture and molecular detection methods

Carla Rodrigues[1*], Kathrin Hauser [2], Niamh Cahill[3], Małgorzata Ligowska-Marzęta[4], Gabriella Centorotola[5], Alessandra Cornacchia[5], Raquel Garcia Fierro[6], Marisa Haenni[6], Eva Møller Nielsen[4], Pascal Piveteau[7], Elodie Barbier[8], Dearbháile Morris[3], Francesco Pomilio[5], Sylvain Brisse[1*]

[1] Institut Pasteur, Université de Paris, Biodiversity and Epidemiology of Bacterial Pathogens, Paris, France;

[2] Institute for Medical Microbiology and Hygiene, Austrian Agency for Health and Food Safety, Vienna/Graz, Austria;

[3] Antimicrobial Resistance and Microbial Ecology Group, School of Medicine, National University of Ireland Galway, Ireland;

[4] Statens Serum Institut, Copenhagen, Denmark;

[5] Istituto Zooprofilattico Sperimentale dell'Abruzzo e del Molise "G. Caporale", Teramo, Italy;

[6] Unité Antibiorésistance et Virulence Bactériennes, Université Claude Bernard Lyon 1 - ANSES, Lyon, France;

[7] INRAE, UR OPPALE, Rennes, France;

[8] Agroécologie, AgroSup Dijon, INRAE, Université Bourgogne Franche-Comté, Dijon, France

***Corresponding authors**:

Sylvain Brisse

Institut Pasteur,

Biodiversity and Epidemiology of Bacterial Pathogens,

28 rue du Docteur Roux, F-75724 Paris, France.

E-mail: sylvain.brisse@pasteur.fr

Carla Rodrigues

Institut Pasteur,

Biodiversity and Epidemiology of Bacterial Pathogens,

28 rue du Docteur Roux, F-75724 Paris, France.

E-mail: carla.parada-rodrigues@pasteur.fr

Contents

Figure S1. Productivity (P_R) results for the three media considered using a reference panel of 50 *Klebsiella* spp. and closely related species.

Some strains (n=5, #22; #26, #35, #46 and #50) showed the same results at least for two media, resulting in only two visible points instead of three.

Figure S2. Evaluation of different protocols for recovery of *Klebsiella* spp. from food matrices.

Figure S3. Protocol to define the optimal temperature of incubation of SCAI medium plates for the recovery of *Klebsiella* spp. from food matrices.

Figure S4. Antibiotic resistance of the *K. pneumoniae* species complex isolates recovered by sample type and by country.

Upper panel: The x-axis represents the number of KpSC resistant isolates for each of the antimicrobials tested and percentages (%) displayed on the graph were calculated based on the total number of isolates.

Lower panel: The x-axis represents the number of KpSC resistant isolates for each of the antimicrobials tested and percentages (%) displayed on the graph were calculated based on the total number of isolates in each country.

Figure S5. Geographic distribution of the common genotypes found among *K. pneumoniae* species complex recovered from food products.

Table S1. Selectivity results for the two media considered using a reference panel of 7 non-*Klebsiella* spp. species.

Table S2. Comparison between the recovery rates of *K. pneumoniae* species complex using two different incubation temperatures of SCAI medium.

Table S3. Chicken meat and salad samples analyzed in the different European countries for the presence of *K. pneumoniae* species complex and its epidemiologic characteristics.

Table S4. Number of *K. pneumoniae* species complex resistant isolates for each of the antimicrobials tested distributed by sampling source and by country.

Table S5. Multidrug resistant strains from chicken meat and salads, sequence-type (ST), phenotypic resistance and identified AMR genes based on WGS.

Table S6. Characteristics of the 131 *K. pneumoniae* species complex isolates recovered: population structure, surface polysaccharides, antimicrobial susceptibility, antimicrobial resistance genes, virulence genes, metal tolerance gene clusters and plasmid replicons.

Table S7. Bacterial reference strains used for agar media performance comparison.
Strains used for the different evaluation criteria are indicated. The correspondent strain code for productivity results in Figure S1 is indicated.

Please see the separate Excel file for access to the supplementary tables.

Figure S1. Productivity (P_R) results for the three media considered using a reference panel of 50 *Klebsiella* spp. and closely related species.

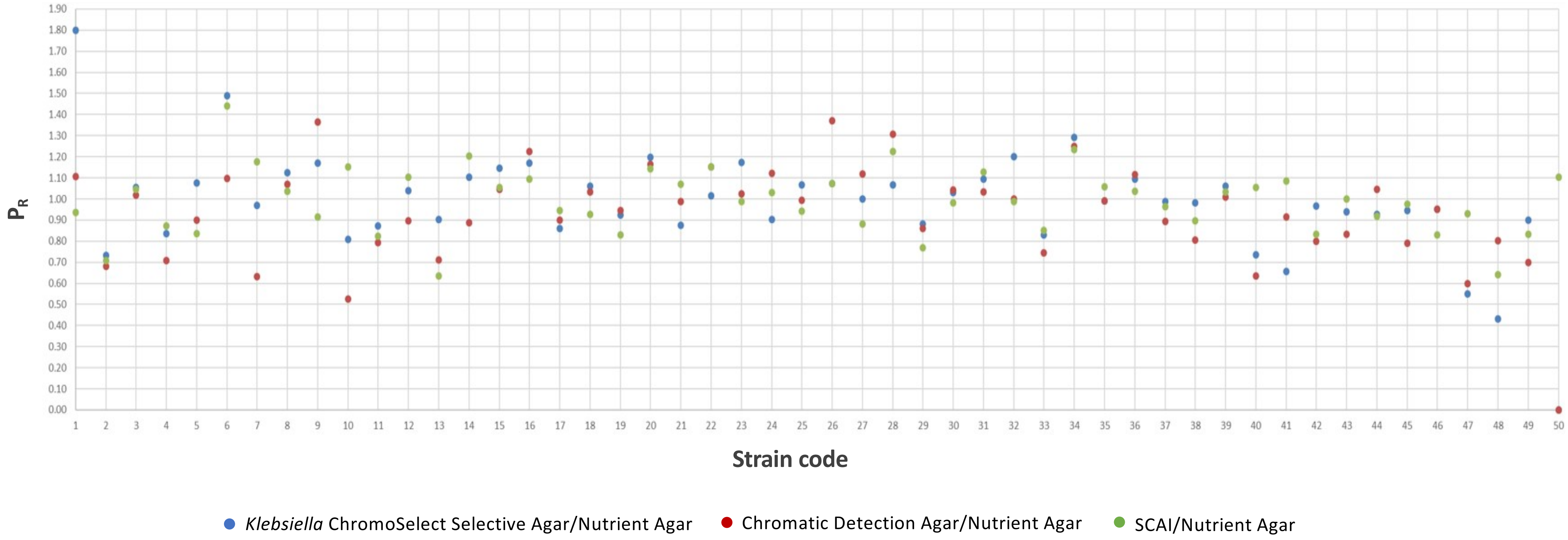

Figure S2 – Evaluation of different protocols for recovery of *Klebsiella* spp. from food matrices.

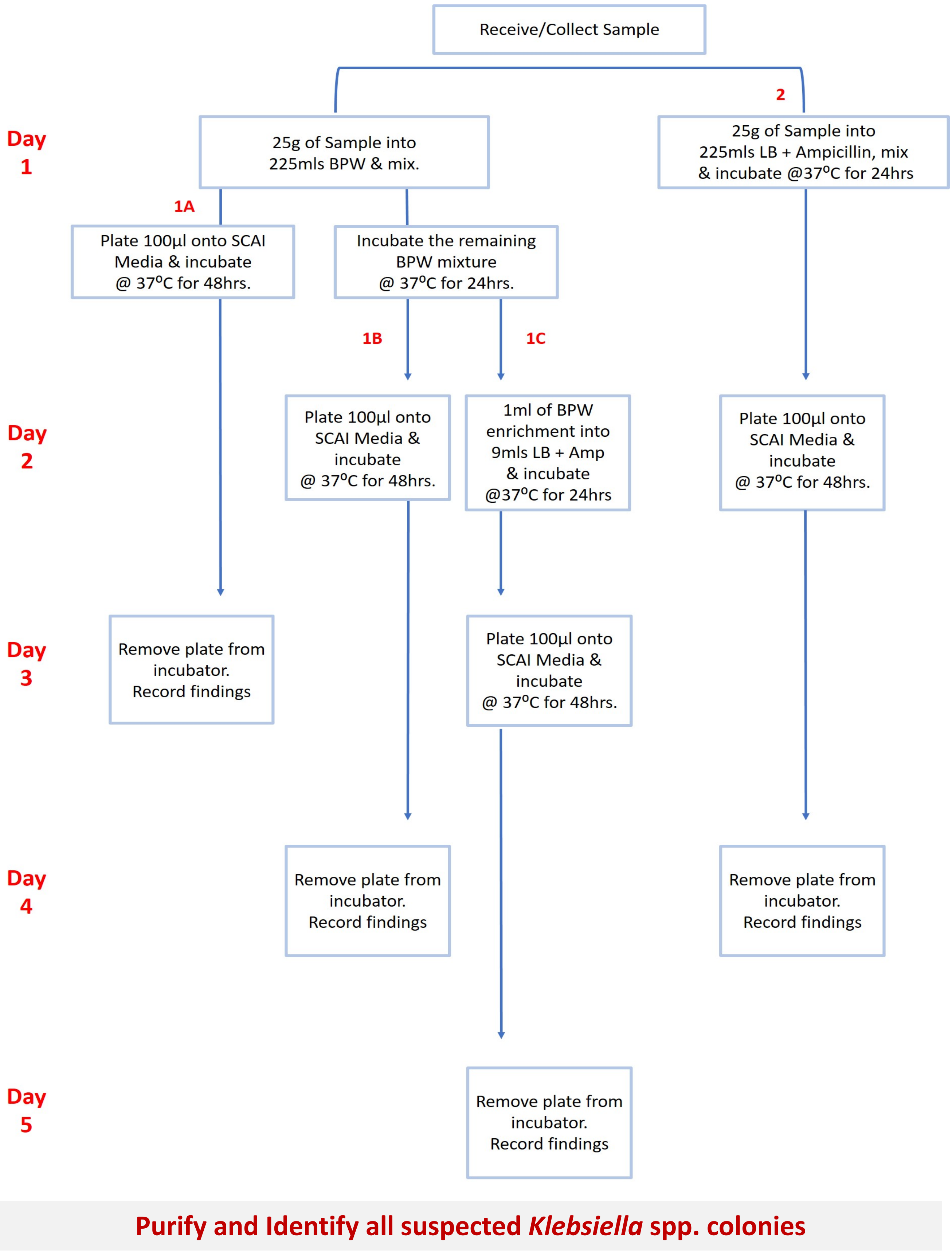

BPW, Buffered Peptone Water
SCAI, Simmons Citrate Agar with Inositol
LB, Lysogeny broth
Amp, Ampicillin
@, at

Figure S3 - Protocol to define the optimal temperature of incubation of SCAI medium plates for the recovery of *Klebsiella* spp*.* from food matrices.

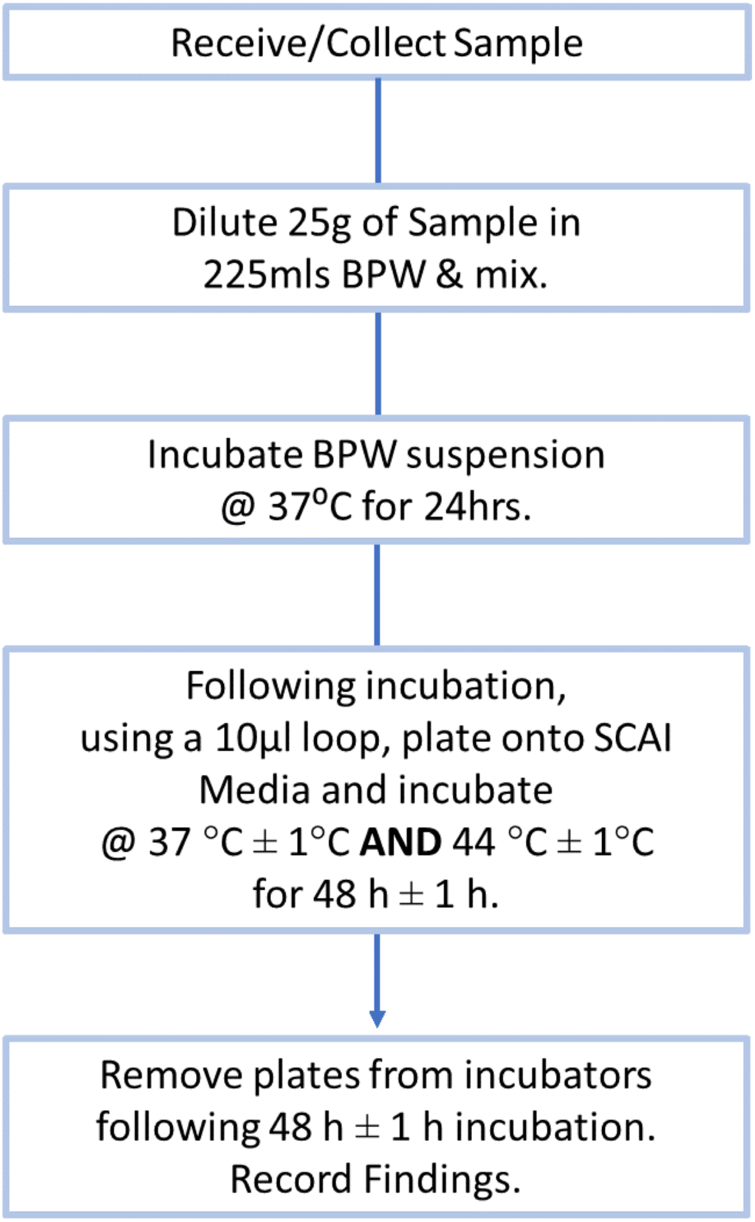

Purify and Identify all suspected *Klebsiella* spp. colonies

BPW, Buffered Peptone Water
SCAI, Simmons Citrate Agar with Inositol
@, at

Figure S4. Antibiotic resistance of the *K. pneumoniae* species complex isolates recovered by sample type and by partner.

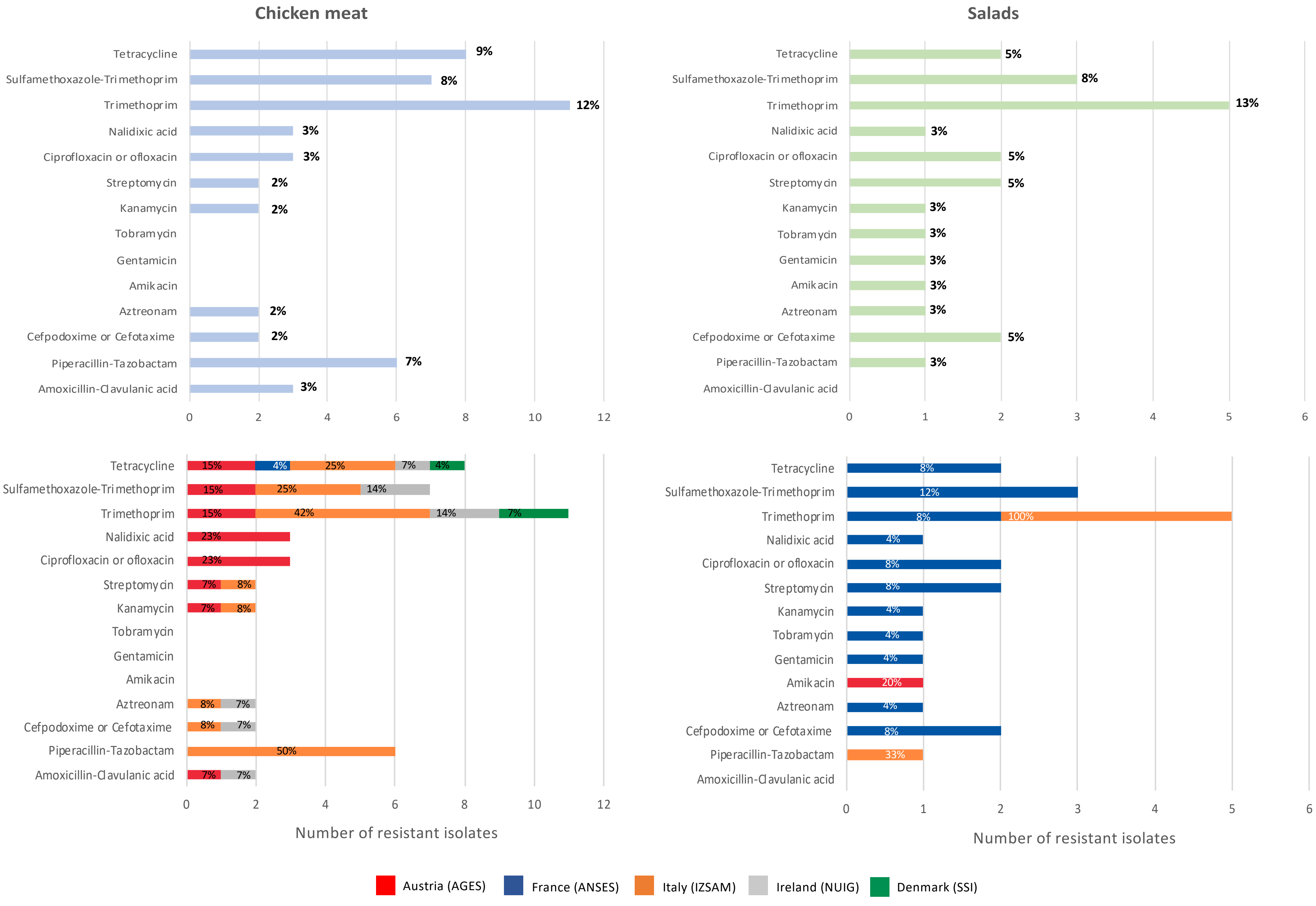

Figure S5. Geographic distribution of the common genotypes found among *K. pneumoniae* species complex recovered from food products.

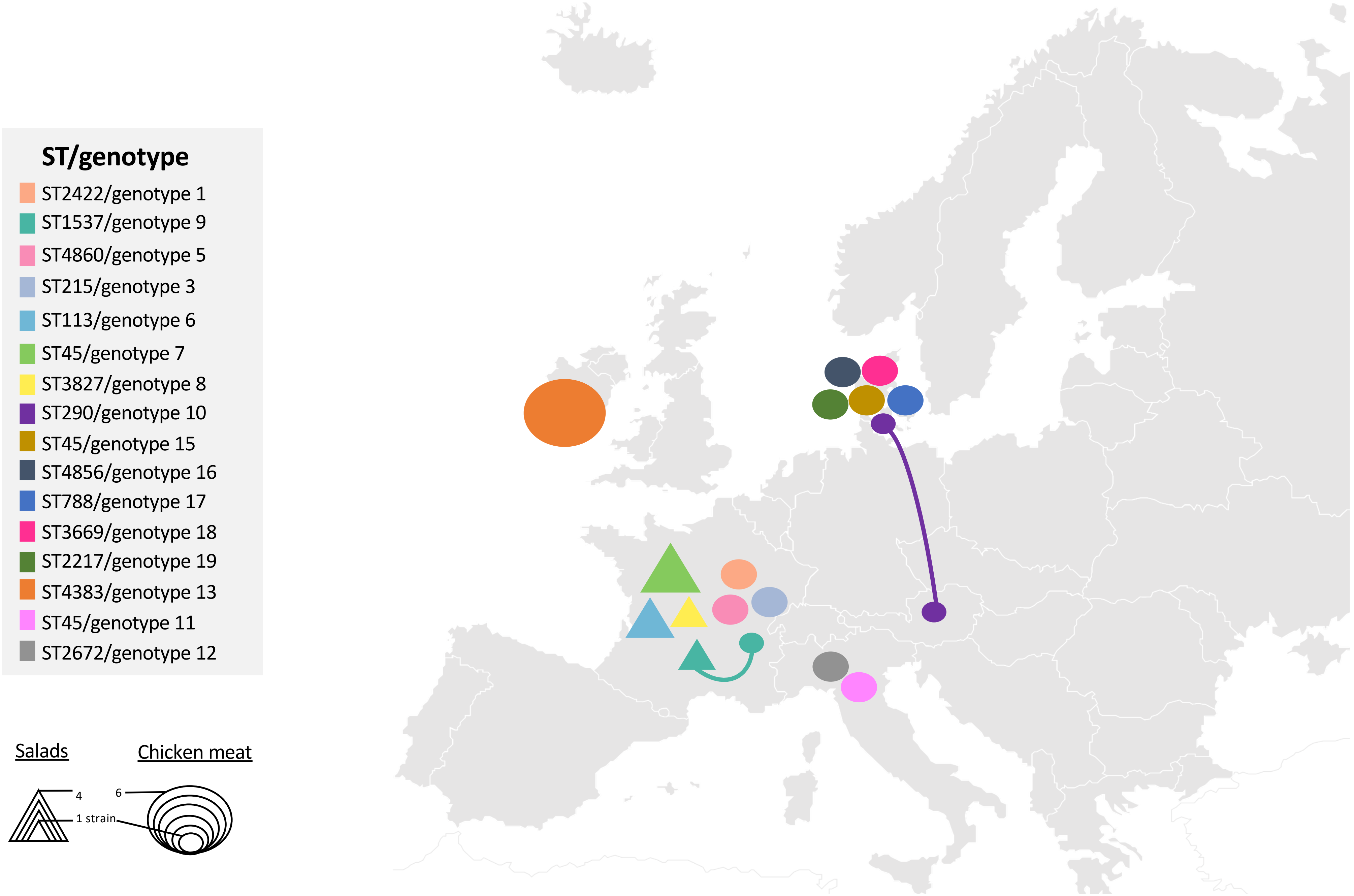

Supplement: SUPPLEMENTAL FILE 2 — Supplemental material. Download SPECTRUM02376-21_Supp_2_seq8.pdf, PDF file, 1.8 MB [file spectrum02376-21_supp_2_seq8.pdf]
